# Revisiting the Molecular Mechanism of Neurological Manifestations in Antiphospholipid Syndrome: Beyond Vascular Damage

**DOI:** 10.1155/2014/239398

**Published:** 2014-03-13

**Authors:** M. Carecchio, R. Cantello, C. Comi

**Affiliations:** ^1^Section of Neurology, Department of Translational Medicine, University of Eastern Piedmont, “Amedeo Avogadro”, Via Solaroli 17, 28100 Novara, Italy; ^2^Interdisciplinary Research Center of Autoimmune Diseases (IRCAD), University of Eastern Piedmont, “Amedeo Avogadro”, Via Solaroli 17, 28100 Novara, Italy

## Abstract

Antiphospholipid syndrome (APS) is a multiorgan disease often affecting the central nervous system (CNS). Typically, neurological manifestations of APS include thrombosis of cerebral vessels leading to stroke and requiring prompt initiation of treatment with antiplatelet drugs or anticoagulant therapy. In these cases, alterations of the coagulation system at various levels caused by multiple effects of antiphospholipid antibodies (aPL) have been postulated to explain the vascular damage to the CNS in APS. However, several nonvascular neurological manifestations of APS have progressively emerged over the past years. Nonthrombotic, immune-mediated mechanisms altering physiological basal ganglia function have been recently suggested to play a central role in the pathogenesis of these manifestations that include, among others, movement disorders such as chorea and behavioral and cognitive alterations. Similar clinical manifestations have been described in other autoimmune CNS diseases such as anti-NMDAR and anti-VGCK encephalitis, suggesting that the spectrum of immune-mediated basal ganglia disorders is expanding, possibly sharing some pathophysiological mechanisms. In this review, we will focus on thrombotic and nonthrombotic neurological manifestations of APS with particular attention to immune-mediated actions of aPL on the vascular system and the basal ganglia.

## 1. Introduction

Clinical manifestations of antiphospholipid syndrome (APS) encompass a wide variety of symptoms that can involve multiple organs at different times over the course of the disease, including the central nervous system (CNS). According to the current clinical diagnostic criteria for APS [1], one or more episodes of arterial, venous, or small vessel thrombosis in any tissue or organ must be demonstrated by means of appropriate imaging studies or histopathology or pregnancy morbidity must be present in the patient's clinical history.

Neurological disturbances due to vascular damage of the CNS are well known in APS and stroke can be the presenting feature of the disease; however, in several patients showing neurological symptoms, no evidence of vascular injury can be detected in the CNS even after extensive imaging investigations. In these patients, mechanisms other than vascular damage have been postulated to play a pathogenetic role in the development of neurological manifestations. At present, however, the only neurological manifestation that satisfies the diagnostic criteria for APS remains cerebral ischemia.

Clinicians should be aware of the constellation of neurological symptoms associated with APS, in order to avoid any diagnostic delay and promptly start an appropriate treatment.

In this paper, we will review the main molecular mechanisms underlying the pathogenesis of vascular and nonvascular damage leading to cerebral dysfunction and neurological manifestations in APS.

## 2. Neurological Manifestations in APS

In 1983, in the original description of the syndrome, Hughes pointed out the importance of cerebral involvement in patients with APS, including cerebrovascular accidents and myelitis [2].

Since then, a number of neurological manifestations have been repeatedly reported in association with primary and secondary APS, sometimes in the absence of stroke, raising the hypothesis that aPL may exert a direct pathogenetic role in the CNS by mechanisms that go beyond vascular thrombosis.

Stroke and transient ischemic attacks (TIA) are the most common manifestation of APS that is an important cause of juvenile stroke, with an age at presentation that is a decade younger than the typical population with classical cardiovascular risk factors [3]. The presence of antiphospholipid antibodies (aPL) in a cohort of 356 unselected patients from a neurological clinic was found to be 15%, with 45% of aPL-positive patients presenting stroke and 13% of cases presenting TIA [4]. In this cohort, the medial cerebral artery was the site most commonly affected by thrombosis, but any brain region can be interested by vascular thrombosis in APS [5, 6]. A recent comprehensive review of the available data from the literature [7] estimated a prevalence of 13.5% of aPL in patients with stroke/TIA, although it is difficult to establish with sufficient confidence the true prevalence of aPL in these patients and in the general population because of different methods used to detect aPL in previously published studies.

Recurrent stroke in patients with aPL has been reported, with no differences between primary and secondary APS [8] and evidence of shortest time to subsequent ischemic event in patients with highest titers of anticardiolipin (aCL) antibodies [3]. Data on the relationship between the recurrence rate of stroke and the levels of aCL antibodies, however, are controversial, with some studies failing to clearly demonstrate this association (see [9] for review).

Cerebral venous thrombosis (CVT) is not a common manifestation of APS, because of a more selective vulnerability of the arterial system to thrombotic events. CVT occurs at young age with more extensive superficial and deep cerebral venous system involvement in aPL-positive patients as compared to negative ones [10]. aPL has been suggested to act as a risk factor for CVT in association with concomitant additional risk factors that predispose to thromboembolism, such as protein C resistance and Leiden's factor V [11].

Recurrent stroke in APS can lead to multifocal damage to the CNS and cause multi-infarct dementia, reported for the first time in 1987 [12].

However, in a case-control study, dementia similar to Alzheimer's disease has been described in 6% of elderly subjects, who were found to have elevated aCL IgG titers without features of autoimmune disease [13]. Subtle cognitive dysfunction such as attention and memory deficit and difficulty in concentration has also been described in patients with aPL without ischemic lesions of the brain (see [9] for review). Cognitive dysfunction has been mainly reported in patients with systemic lupus erythematosus (SLE) and APS and persistently elevated IgG and IgA aCL levels were associated with reduced psychomotor speed and executive dysfunctions [14].

Movement disorders are known to be part of the clinical spectrum of APS.

Chorea is a hyperkinetic movement disorder characterized by involuntary, patternless, purposeless movement continuously flowing from a body part to another in an unpredictable way.

Several studies have established a relationship between chorea and aPL, especially in APS secondary to SLE, in which chorea has been described in 58% of cases in a previously published series by Cervera et al. [15], and the prevalence of aPL in patients with lupus chorea has been reported in up to 92% of patients [15–17]. Chorea is also the most commonly encountered movement disorder in primary APS, with an estimated frequency of 1.3% [18]. Since vascular lesions in the basal ganglia are present only in a subset of patients with chorea, a direct binding of aPL to CNS antigens has been postulated [19].

Other less frequent movement disorders previously described in APS include tics [20, 21], corticobasal syndrome [21, 22], hemidystonia [23, 24], paroxysmal nonkinesigenic dyskinesias [25], parkinsonism [26], cerebellar ataxia [27], and complex mixed presentations [28]. For a more comprehensive review on movement disorders in SLE and primary APS, see Baizabal-Carvallo [29].

Nonthrombotic neurological manifestations occur in up to 40% of patients with elevated aPL [4] and include seizures, headache, transverse myelitis, sensorineural hearing loss, multiple sclerosis, and peripheral neuropathy including Guillain-Barrè syndrome [30].

Seizures in APS are present in about 10% of patients [31] and either can be secondary to vascular lesions or occur without evidence of previous stroke. In this regard, a functional interaction between aPL and neuronal membranes has been demonstrated* in vitro* by inducing depolarization of synaptoneurosomes by means of IgG from APS patients. This has been suggested to cause epilepsy in APS, and an association between serum levels of aPL and seizure occurrence has been described [32]. On the other hand, stroke and cerebrovascular disease have been indicated in different studies as an independent risk factor for developing seizures in APS [31, 33].

Transverse myelitis (TM) is a rare manifestation of APS with an estimated prevalence of <1%; it is characterized by acute inflammation affecting mainly the thoracic segment of the spinal cord that causes sensory loss below the level of the lesion, motor, and sphincter disturbances [34]. Spinal cord MRI generally shows lesions exceeding three vertebral segments in length with central involvement, but shorter lesions can be detected [35]. In SLE, a strong association between aPL positivity and TM has been reported [36]; however, the pathophysiology of TM is still controversial; vascular injury secondary to vasculitis, hypercoagulable state, and a direct role of antineuronal antibodies have all been postulated [34].

With regard to the peripheral nervous system, its involvement in APS can be present either in the form of* mononeuritis multiplex* (often caused by vasculitis associated with SLE and other immunorheumatological diseases) or as peripheral, mainly axonal, neuropathy that is often asymptomatic and revealed only by means of electrophysiological studies [34].

Thrombotic and nonthrombotic neurological manifestations in APS are listed in Table 1.

## 3. Thrombosis in APS: Pathogenetic Mechanisms

Many studies have shown that aPL are pathogenetic in APS and play a prothrombotic role both in the arterial and in the venous vascular system [38, 39]. At present, the most likely hypothesis to explain this phenomenon is that these antibodies exert multiple actions at different levels ultimately leading to alteration of the coagulation system in a prothrombotic manner.

The predominant antibodies in APS are directed against protein antigens that bind to anionic phospholipids, such as Beta 2-glycoprotein I (B2GPI) and prothrombin. Prothrombin is a proenzyme cleaved by the prothrombinase complex and subsequently leads to the generation of thrombin. B2GPI is a plasma protein consisting of four domains (from I to IV) of approximately 60 amino acids and one domain (domain V) of 84 amino acids, through which binding to anionic phospholipids takes place [40]. Antibodies against B2GPI seem to display different domain specificity; in particular, antibodies recognizing domain I, described as highly immunogenic, represent most of the anti-B2GPI antibodies and have been detected in many patients with a history of thrombosis, whereas antibodies directed against domain V do not seem to be associated with thrombosis [41]. aPL bind to an epitope present in domain I that is normally not available to binding because of the physiological circular conformation of B2GPI. Under specific conditions, such as infections, the protein conformation can change resulting in exposure of the immunogenic epitope of domain I, to which autoantibodies bind [38].

Platelet factor 4 (PF4) binds to dimerized B2GPI, and this complex is recognized by anti-B2GPI antibodies. The PF4-B2GPI complex activates platelets by p38 MAP kinase phosphorylation, GPIIb/3a receptor expression, and thromboxane B2 synthesis and release, thus leading to platelets activation and thrombosis [42]. Other mechanisms of platelet activation have been described, including the interaction between B2GPI-recognizing antibodies and the glycoprotein Ib- (GPIb-) apolipoprotein receptor E2 (APOER2) complex and the binding of B2GPI/anti-B2GPI complexes through LDL receptor-related proteins [43–46].

The hypercoagulable state promoted by aPL is thought to be based on several other mechanisms. In fact, aPL induce the overproduction of tissue factor (TF), thus activating the extrinsic pathway of the coagulation, increase resistance to activated protein C (APC), and bind to annexin A5 (AnxA5). This protein binds to the exposed negatively charged phospholipids on the surface of the cell making them unavailable to other coagulation factors and thus exerting an anticoagulant activity. Antiphospholipid antibodies disruption of annexin A5 anticoagulant shield is thought to be an important thrombogenic mechanism [47].

Endothelial dysfunction is an additional important mechanism underlying thrombotic events in APS. aPL can in fact induce a procoagulant activity in endothelial cells (EC) by inducing expression of procoagulant, proinflammatory, and proadhesive molecules that favors the binding of circulating leukocytes. After aPL binding, EC increase the expression of adhesion molecules (ICAM-1, VCAM-1, and E-selectin) [48], upregulate tissue factor transcription through p38 MAP kinase phosphorylation and the NF-*κ*B pathway [49, 50], and increase cytokines/chemokines production (IL-4, IL-6, IL-8, MCP-1, and TNF-*α*) [51–54].

Finally, a possible pathogenetic role of the complement system in APS-associated thrombosis has been suggested in animal models of mice deficient for the complement factors C3 and C5 that do not show thrombosis or pregnancy loss induced by aPL [55, 56]. Moreover, elevated levels of complement activation products have been detected in plasma of patients with APS who had a cerebral ischemic event as compared to APS patient without ischemia [57].

## 4. Nonthrombotic Manifestations in APS

Beside vascular lesions in the CNS and the underlying thrombotic mechanisms, a direct effect of aPL in the CNS has been postulated to explain neurological symptoms without evidence of cerebral stroke. Data from animal models largely support this hypothesis.

Mice immunized with the main APS autoantigen, B2GPI, show behavioral changes such as higher stair-climbing activity and anxiety/exploratory behavior as compared to adjuvant-immunized mice in a staircase apparatus [58]; similar results have been obtained in mice by immunization with a pathogenic monoclonal antibody [59]. In both experimental APS models, no vascular damage to the CNS could be detected.

Passive immunization of mice with B2GPI as well as intraventricular injection of IgG aPL purified from patients' serum leads to memory and learning deficits more evident 4-5 months after single immunization [60, 61] with evidence of antibodies binding to specific areas of the brain, including hippocampus, cortex, and choroid plexus.

More recently, Katzav et al. demonstrated that, in a mouse model of Alzheimer's disease carrying the APP 695 SWE mutation, immunization with B2GPI was associated with mature amyloid plaques deposition on a female background and the development of motor hypoactivity and impaired cognition. The long-term exposure to elevated aPL levels of susceptible brains would therefore seem to induce AD pathology. The authors propose that APP, which also plays a role as coagulation inhibitor, is affected in APS [62] and that this leads to exposure of the brain to high levels of thrombin, that in turn causes consumption of coagulation inhibitors such as APP, and subsequent upregulation of APP synthesis that may determine an increased risk of developing Alzheimer's pathology [63].

Another possible link between aPL and cognitive dysfunction would be the increased release of proinflammatory cytokines. Indeed, it is well documented that, in neurodegenerative diseases characterized by cognitive dysfunction and in particular in AD, proinflammatory cytokines play a significant pathogenetic role [64, 65].

The pathogenesis of movement disorders, especially chorea, in APS has recently been a matter of debate. The first hypothesis to explain involuntary movements in APS was the involvement of lenticulostriate arteries in thrombotic processes producing ischemia of the basal ganglia. However, basal ganglia lesions in APS-related chorea have been rarely reported [15]. Despite the technical limitations of imaging studies that might have overlooked small-vessel thrombosis in the basal ganglia, alternative pathogenetic mechanisms have been proposed to explain movement disorder in APS, based on recent works that demonstrated a direct binding of aPL to cells in the CNS.

How aPL and lymphocytes may gain access to the CNS is still not completely understood. aPL may enter the CNS by causing an alteration of the endothelial cells, as previously discussed, ultimately leading to endothelial dysfunction [66, 67]. This may cause a disruption of the blood-brain barrier (BBB) that, when intact, prevents antibodies and lymphocytes from accessing the CNS. In case of disruption or altered permeability of the BBB, aPL may be able to cross it and react with specific autoantigens expressed by neurons, astrocytes, and other CNS cells. Moreover, resting lymphocytes cannot cross the BBB, but activated lymphocytes can cross it and expand in the CSF if the autoantigen is encountered. After this initial phase, aPL may exert a direct pathogenetic role by direct neuronal binding. In this regard, the ability of aPL to bind to neuronal cell surface has been previously demonstrated in several publications. Caronti et al. [68] showed that in experimental animal models anti-B2GPI antibodies can bind to various cells in the CNS, including neurons, astrocytes, and endothelial cells. More recently, Dale et al. [69] demonstrated the presence of IgG antibodies binding to neuronal cell surface antigens in four paediatric patients with APS and chorea. The authors used a neuronal cell line with dopaminergic features, suggesting that an autoimmune mediated mechanism causing functional alterations of the basal ganglia circuitry may be the underlying pathogenetic mechanism in APS-related chorea. Interestingly, aPL have been demonstrated to depolarize synaptic brain rat extracts [32] and may exert a similar function in humans. A direct binding of autoantibodies to antigens expressed in the basal ganglia has been detected in patients with autoimmune encephalitis characterized by various combinations of movement disorders (chorea, dystonia, Parkinsonism, and tics) and psychiatric disturbances. The authors detected IgG antibodies directed against dopamine D-2 receptors in the sera of patients suffering from basal ganglia encephalitis, a type of encephalitis that can affect both children and adults [70].

Several autoantibodies have been detected in the past years in autoimmune encephalitis, both in primary and in paraneoplastic cases [71]. In this scenario, anti-B2GPI antibodies in APS may represent a further addition to the already long list of antibodies directed against various CNS antigens expressed either on the neuronal surface or at an intracellular level.

Even if the exact antigenic specificity of aPL in the CNS is still to be unraveled, an immunological mechanism may explain the biological basis of chorea in APS, similarly to other autoimmune movement disorders.

In fact, in the past years, different autoimmune basal ganglia disorders presenting with movement disorders and neuropsychiatric disease have been described [72]. Historically, the prototype of movement disorder caused by an autoimmune process was represented by Sydenham's chorea, characterized by chorea, behavioral alterations, dysarthria, and, less commonly, hypotonia (chorea paralytica). Sydenham's chorea is thought to be caused by a molecular mimicry mechanism following a streptococcal infection, but, despite accumulating evidence pointing to an immune-mediated mechanism involving the basal ganglia, no definite pathogenic autoantibodies have been detected to date. Antibodies against lysoganglioside GM1 and tubulin [73] and against intracellular antigens [74, 75] have been detected with various techniques, but no definitive conclusion on their pathogenic role in the development of clinical symptoms can be drawn.

The hypothesis that streptococcal or other infections could trigger basal ganglia functional alterations manifesting with different combinations of movement disorders and neuropsychiatric symptoms, especially tics and obsessive-compulsive behaviour, was explored in the nineties, and in 1998 the term PANDAS (pediatric autoimmune neuropsychiatric disorders associated with streptococcus) was coined [76]. Similarly to Sydenham's chorea, antibodies against lysoganglioside GM1 have been detected in some PANDAS patients [77], while other studies failed to identify antibodies against neuronal antigens in cultured neuron-like cells or by means of other techniques [78–81]. Hence, several controversies are still present in the clinical and etiological definition of PANDAS and its overlap with other waxing and waning tic disorders such as Tourette's disease [72].

Within the spectrum of immune-mediated basal ganglia diseases presenting with psychiatric symptoms and movement disorders, two additional entities are worth mentioning. The first one is NMDAR receptor encephalitis, described for the first time as a paraneoplastic encephalitis in female patients with ovarian teratoma [82] and later in children and adolescents previously labeled as being affected by encephalitis lethargica [83, 84]. In these subjects, antibodies against an extracellular epitope located in the N-terminal domain of the NR1 subunit of the N-methyl-D-aspartate receptor were found to cause chorea and other hyperkinetic movement disorders including dystonia, stereotypy, hemiballismus, orofacial and limb dyskinesias, oculogyric crises, and catatonic phenomena (waxy flexibility), as well as rigidity in the later stages, often presenting in complex combinations and associated with anxiety, agitation, and psychosis [72, 85]. It has been suggested that a decrease of NMDA receptors in inhibitory GABAergic neurons and glutamatergic synapses causes disinhibition of excitatory pathways and an increase in extracellular glutamate, resulting in a frontostriatal syndrome (psychosis, catatonia, mutism, rigidity, and dystonia) as well as disinhibition of brainstem central pattern generators causing complex elaborate movements and dyskinesias [86]. Interestingly, aPL have been suggested to interfere with excitatory pathways in glutamatergic rat cerebellar granule cells by a mechanism involving overactivation of the NMDA glutamate receptor [87].

Another recently described autoimmune encephalitis is limbic encephalitis associated with antivoltage gated potassium channel (VGKC) antibodies. Also in this condition, different movement disorders have been described, encompassing chorea, Parkinsonism, tremor, and myoclonus [85]. Anti-VGKC antibodies are now recognized to bind to different components of VGKC complexes such as leucine-rich glioma inactivated-1 (Lgi1) [88] or contactin-associated protein 2 (Caspr2) [89], proteins that are part of transsynaptic complexes and neural cell adhesion molecules involved in modulation of synaptic transmission. Characteristic faciobrachial dystonic seizures have been described prior to the onset of other neurological and psychiatric manifestations of limbic encephalitis due to anti-VGKC antibodies and should be promptly recognized and treated to prevent further damage to the CNS [90].

All these neurological immune-mediated syndromes share some clinical features such as a combination of generally hyperkinetic movement disorders with different degrees of complexity (from subtle chorea to typical upper limb stereotypies in paraneoplastic anti-NMDAR encephalitis associated with ovarian teratoma) and neuropsychiatric symptoms, subacute onset, progressing within days or weeks, and response to steroids, intravenous immunoglobulins, immunosuppressive agents, plasmapheresis, and monoclonal antibodies such as rituximab.

While ischemic manifestations of APS always imply the initiation of either antiplatelet drugs or long-term anticoagulants (warfarin), no standard treatment is available for nonvascular neurological manifestations of APS. First-line treatment of aPL-related chorea generally includes symptomatic antidopaminergic drugs such as haloperidol or dopamine-depleting agents such as tetrabenazine; however, the probable flogistic/autoimmune mechanisms underlying these symptoms justify the use of steroids, especially in case of monotherapy failure. The European League against Rheumatism (EULAR) recommends the use of glucocorticoids or immunosuppressive agents (azathioprine, cyclophosphamide) in severe cases of APS secondary to SLE to control disease activity [91]. Isolated reports on the efficacy of intravenous immunoglobulins, plasmapheresis, and rituximab have been published as well, in cases with unsatisfactory response to conventional drugs [92]. A recent pilot phase II trial of rituximab for noncriteria APS manifestations has been published, and its results suggest that this anti-CD20-monoclonal antibody may be helpful in controlling cognitive dysfunction [93]. However, randomized clinical trials are needed to determine whether B cell depleters such as rituximab or B cell modulators can be effective in treating thrombotic and nonthrombotic manifestations of APS [94].

## 5. Conclusion

Beyond classic thrombotic presentations such as ischemic stroke, several polymorphic neurological manifestations of APS have emerged over the past years, with particular interest for those lacking a clear anatomical correlate on imaging studies. An increasing body of evidence from experimental APS models as well as from patients currently supports an immune-mediated pathogenesis underlying nonthrombotic manifestations of APS such as movement disorders and neuropsychiatric symptoms that clearly indicate a functional involvement of basal ganglia. Moreover, several newly recognized antibodies-mediated neurological diseases have come to light in recent years, such as anti-NMDAR and anti-VGKC encephalitis that partially resemble APS in the type of onset, clinical course, treatment options, and possible triggering factors such as infections. All these complex and multifaceted basal ganglia autoimmune diseases represent an upcoming challenge for clinicians, who need to promptly recognize and effectively treat them in early stages, in order to avoid diagnostic and therapeutic delay that may lead to a more extensive spread of the autoimmune aggression and damage to the CNS.

## Figures and Tables

**Table 1:** Neurological manifestations of APS.

Neurological manifestations associated with antiphospholipid syndrome	Features
Thrombotic manifestations	
Spinal cord stroke	Uncommon feature; less frequent than transverse myelitis
Acute ischemic encephalopathy	Uncommon feature in APS secondary to SLE; presents with tetraparesis, confusion, and hyperreflexia
Ischemic stroke Transient ischemic attack (TIA)	The most common manifestations of APS; important cause of juvenile stroke; any brain region can be interested
Cerebral venous thrombosis	Uncommon vascular manifestation

Nonthrombotic manifestations	
Headache	Frequent and often untreatable; no definite association between aPL positivity and type of headache [34]
Multiple sclerosis	APS can mimic multiple sclerosis; no definite tests to differentiate these entities are available
Transverse myelitis	Rare acute inflammatory manifestation; more common in APS secondary to SLE
Sensorineural hearing loss	Acute onset in the presence of aPL may be a manifestation of APS [37]
Guillain-Barrè syndrome	Antiphospholipid antibodies probably produced as a result of myelin damage
Peripheral neuropathy	*Mononeuritis multiplex* due to vasculitis is most commonly found in SLE; axonal neuropathy can be asymptomatic in PAPS
Cognitive dysfunction and dementia	Caused by multiple brain strokes; Alzheimer's disease-like dementia in 6% of cases
Idiopathic intracranial hypertension	Can be the presenting feature of APS
Epilepsy	In 10% of patients; primary or secondary to stroke
Chorea and other movement disorders	Rarely due to stroke in the basal ganglia; frequent in patients with APS secondary to SLE
